# A Multicenter Pilot Randomized Controlled Trial of a Digital Symptom Management Platform (WECARE) for Gastric Cancer Survivors

**DOI:** 10.3390/cancers18091329

**Published:** 2026-04-22

**Authors:** Geum Jong Song, Jae-Seok Min, Rock Bum Kim, Ki Bum Park, Bang Wool Eom, Jong Hyuk Yun, Hoon Hur, Jeong Ho Song, Hayemin Lee, Su Mi Kim, Eun Young Kim, Hyungkook Yang, Joongyub Lee, Sang-Ho Jeong

**Affiliations:** 1 Department of Surgery, Soonchunhyang University Cheonan Hospital, Soonchunhyang University College of Medicine, Cheonan 31151, Republic of Korea; gjsong@schmc.ac.kr (G.J.S.); 109206@schmc.ac.kr (J.H.Y.); 2 Department of Surgery, Korea University College of Medicine, Seoul 02841, Republic of Korea; mdoogy24@korea.ac.kr; 3 Division of Foregut Surgery, Korea University Anam Hospital, Seoul 02841, Republic of Korea; 4 Regional Cardiocerebrovascular Disease Center, Gyeongsang National University Hospital, Jinju 52828, Republic of Korea; 5 Department of Surgery, School of Medicine, Kyungpook National University, Kyungpook National University Chilgok Hospital, Daegu 41404, Republic of Korea; 6 Center for Gastric Cancer, National Cancer Center, Goyang 10408, Republic of Korea; 7 Department of Surgery, Ajou University School of Medicine, Suwon 16499, Republic of Korea; 8 Division of Gastrointestinal Surgery, Department of Surgery, Bucheon St. Mary’s Hospital, College of Medicine, The Catholic University of Korea, Bucheon-si 14647, Republic of Korea; hymin@catholic.ac.kr; 9 Department of Surgery, CHA Bundang Medical Center, CHA University, Seongnam 13488, Republic of Korea; 10 Department of Surgery, Uijeongbu St. Mary Hospital, College of Medicine, The Catholic University of Korea, Uijeongbu-si 11765, Republic of Korea; 11 Lunit CARE Inc., Seoul 06241, Republic of Korea; 12 Department of Preventive Medicine, Seoul National University College of Medicine, Seoul 03080, Republic of Korea; 13 Department of Surgery, Gyeongsang National University College of Medicine and Gyeongsang National University Changwon Hospital, Changwon 51471, Republic of Korea

**Keywords:** Stomach neoplasms, postgastrectomy syndromes, telemedicine, quality of life, patient-reported outcome measures

## Abstract

**Background:** Gastric cancer survivors frequently encounter a “care gap” after discharge because of complex postgastrectomy syndromes. We evaluated “WECARE,” a bidirectional digital health platform designed to provide real-time symptom monitoring and multidisciplinary support. The primary goal of this study was to assess the efficacy of the platform by measuring the change in the Korean Quality of Life Questionnaire for Gastric Cancer Survivors (KOQUSS-40) total score over a six-month recovery period. **Methods:** This nationwide, multicenter pilot randomized controlled trial was conducted by the Korean Quality of Life in Stomach Cancer Patients Study Group (KOQUSS) across nine tertiary centers in Korea. A total of 88 patients who underwent curative gastrectomy were enrolled. Following an initial optimization phase involving 22 patients, the remaining 66 patients were randomized at a 1:1 ratio to the WECARE group or the control group. The WECARE group used a platform integrating the KOQUSS-40 algorithm for structured symptom reporting, real-time feedback on nutrition and exercise, and educational content on meal planning, symptom coping, and recovery. Assessments were performed at baseline and at 1, 3, and 6 months after discharge. **Results:** The WECARE group showed high feasibility and acceptability, with an adherence rate of 86.7% and an 82% satisfaction rate. At 6 months, the KOQUSS-40 total score (primary endpoint) did not differ significantly between the WECARE and control groups (85.3 ± 1.6 vs. 83.8 ± 1.6, *p* = 0.603). However, the WECARE group showed a numerically favorable recovery trajectory from the acute postoperative phase. Subgroup analysis revealed a positive trend in reflux symptom management in the WECARE group (*p* = 0.0856). In addition, more than 77% of users reported that the platform improved their self-management capabilities. **Conclusions:** The WECARE platform is a feasible and acceptable digital intervention for gastric cancer survivors. Although the primary endpoint was not significantly different, the favorable recovery trajectory, high adherence, and patient engagement support further evaluation in larger studies with longer follow-up and broader healthcare settings.

## Simple Summary

Many gastric cancer survivors experience persistent symptoms after surgery, including reflux, dumping syndrome, and difficulty maintaining proper nutrition. Although hospital stays have become shorter, many patients still need support after discharge. We developed WECARE, a digital symptom management platform that allows patients to report symptoms and receive tailored feedback on diet, activity, and recovery. In this multicenter pilot randomized trial, we evaluated whether WECARE could improve recovery-related quality of life during the first 6 months after gastrectomy. Although the overall quality-of-life score did not significantly differ from standard care, patients using WECARE showed a favorable recovery trajectory, high adherence, and strong satisfaction. These findings suggest that digital health platforms may support self-management and continuity of care for gastric cancer survivors after surgery.

## 1. Introduction

Advancements in surgical techniques and perioperative management have significantly shortened hospital stays for cancer patients, yet the transition from hospital to home remains a vulnerable period often referred to as the “care gap.” [1]. During this critical phase, patients frequently encounter complex physical and psychological challenges in the absence of immediate clinical supervision. Recent studies in various malignancies have shown that digital health interventions can effectively bridge this gap by providing continuous symptom monitoring and psychological support [2,3,4,5]. For instance, in lung and colorectal cancer, smartphone-based monitoring has been shown to improve overall survival and reduce the number of emergency department visits by enabling early detection of complications. However, despite the high prevalence of postgastrectomy syndromes—including dumping syndrome, nutritional deficiencies, and bile reflux—there is a notable paucity of specialized, evidence-based digital platforms designed for the longitudinal care of gastric cancer survivors [6,7]. Current digital tools often remain generic and fail to address the intricate dietary and metabolic needs unique to patients who have undergone gastrectomy [2,3,8].

To address these unmet needs, we developed “WECARE,” a patient-centered digital platform designed to transform the postoperative recovery trajectory through bidirectional interaction. Unlike conventional one-way educational applications, WECARE functions as a dual-purpose ecosystem that allows clinicians to objectively monitor patient-reported outcomes (PROs) in real time while empowering patients to increase their self-management literacy. The platform integrates validated algorithms, such as the KOQUSS-40, to quantify postgastrectomy symptoms, providing patients with immediate, tailored feedback on nutrition, physical activity, and symptom mitigation. By facilitating this continuous data flow, WECARE shifts the paradigm of gastric cancer care from reactive hospital-based visits to proactive, home-based management. This approach not only alleviates the burden on the healthcare system but also fosters patient autonomy, ensuring that care continuity is maintained through a robust, evidence-based digital interface [9,10,11,12].

The primary goal of this study was to assess the efficacy of the platform by measuring the change in the Korean Quality of Life Questionnaire for Gastric Cancer Survivors (KOQUSS-40) total score over a six-month recovery period.

## 2. Methods

### 2.1. Multicenter Study Setting and the KOQUSS Study Group

This multicenter pilot randomized controlled trial evaluated the clinical utility of a digital symptom management platform (WECARE) in patients undergoing gastrectomy for gastric cancer. The detailed study design has been described in a published study protocol [9]. The trial was coordinated by the Korean Quality of Life in Stomach Cancer Patients Study Group (KOQUSS Study Group), a collaborative research network affiliated with the Korean Gastric Cancer Association. Participants were recruited from nine tertiary referral hospitals across South Korea between July 2024 and December 2025. Recruitment was intentionally staged because the study was designed as a pilot trial to assess feasibility, adherence, and preliminary efficacy signals before future large-scale validation. This study was approved by the institutional review board (IRB) of each participating institution, and written informed consent was obtained from all patients prior to enrollment. This trial was registered at ClinicalTrials.gov (NCT06395935).

### 2.2. Eligibility Criteria

Patients were eligible for participation if they were aged between 19 and 75 years, had histologically confirmed gastric cancer, were undergoing planned curative gastrectomy (total, distal, or proximal) for clinical stage I–III disease, had access to the web-based WECARE platform through a smartphone or internet-enabled device, had the ability to understand and complete the study questionnaires, and could provide written informed consent. Patients who were unable to attend scheduled follow-up visits or who had severe psychiatric disorders, significant uncontrolled comorbidities, or communication difficulties that could interfere with participation were excluded. Patients judged unsuitable for the study by the treating surgeon were also excluded.

### 2.3. Randomization and Intervention

This pilot trial was conducted in two phases. Initially, 22 patients were enrolled to evaluate the feasibility of the platform, optimize the workflow, and refine study procedures before randomized evaluation. Because the primary aim was feasibility and preliminary efficacy signal assessment, no formal sample size calculation was performed. After this phase, the remaining 66 participants were randomly assigned in a 1:1 ratio to either the WECARE group or the control group. The patients were randomized using a computer-generated allocation sequence with block randomization stratified by the participating center, prepared by an independent statistician, with allocation concealed through a centralized computer-based randomization system. Because the intervention involved a digital platform, the blinding of participants and clinicians was not feasible. The WECARE group used the web-based platform after surgery. The platform collected patient-reported symptoms using the KOQUSS-40 questionnaire and translated symptom patterns into algorithm-based personalized feedback related to meal frequency and portion size, food selection, reflux and dumping precautions, hydration, physical activity, and guidance on when to seek medical attention. Educational resources were delivered as text, images, and short video materials to reinforce the same symptom-management domains. The bidirectional interaction of the platform was based on repeated patient symptom input and return of symptom-specific feedback, allowing patients to monitor their recovery longitudinally while enabling clinicians to review patient-reported information over time. The control group received standard postoperative follow-up according to institutional practice. Quality-of-life questionnaires were completed during outpatient visits at the same time points as those in the intervention group.

### 2.4. Outcome Measures

The primary outcome of the study was postoperative quality of life, which was measured using the Korean Quality of Life Questionnaire for Gastric Cancer Survivors (KOQUSS-40). This questionnaire is a validated instrument consisting of 40 items evaluating symptoms and functional outcomes after gastrectomy. Assessments were conducted before surgery and at 1, 3, and 6 months after surgery. Secondary outcomes included body weight change, nutritional parameters (hemoglobin level, albumin level, lymphocyte count, and prognostic nutritional index), compliance with questionnaire completion, self-efficacy, physical activity level, and patient satisfaction with the WECARE platform.

### 2.5. Statistical Analysis

Baseline characteristics were compared between the WECARE and control groups to assess the balance achieved through randomization. Continuous variables are presented as the mean ± standard deviation (SD), and categorical variables are presented as the frequency and percentage. For continuous variables, between-group differences were assessed using the Wilcoxon rank-sum test, given the potential for nonnormal distributions in clinical data. For categorical variables, between-group comparisons were performed using Pearson’s chi-square test when expected cell frequencies were sufficient or Fisher’s exact test when expected cell counts were less than 5. The magnitude of between-group differences was quantified using the standardized mean difference (SMD). An SMD of less than 0.1 is generally considered indicative of good balance between groups, whereas an SMD greater than 0.2 may suggest a meaningful imbalance that warrants consideration in subsequent analyses.

Health-related quality of life was assessed using KOQUSS-40, which comprises a total score and 11 domain scores, each transformed to a 0–100 scale (higher scores indicate better QOL). The total score was calculated as the mean score of eight symptom-related domains (indigestion, dysphagia, reflux, dumping syndrome, bowel habit change, constipation, psychological factors, and worry about cancer). Linear mixed-effects models (LMMs) were fitted for the total and domain-specific scores, with group, time point (baseline and 1, 3, and 6 months), and their interaction as fixed effects and a random intercept for each patient. The primary inferential focus was on the between-group difference in longitudinal recovery trajectory over time while accounting for repeated within-patient measurements. Estimated marginal means (EMMs) with 95% CIs were used for between-group comparisons at each time point. Missing data were handled using linear mixed-effects models under the assumption of missing at random. Group-specific slopes over time were estimated using the emtrends function, and between-group differences in trajectories were assessed through pairwise contrasts. Effect sizes were quantified using Cohen’s d (negligible: <0.2, small: 0.2–0.5, medium: 0.5–0.8, large: ≥0.8) and averaged across time points to obtain an overall effect size.

All comparative efficacy analyses were performed in the randomized cohort according to a modified intention-to-treat principle, including all randomized patients who underwent surgery and had at least one post-baseline assessment. The initial 22-patient optimization phase was used to assess feasibility and refine platform procedures and was included in descriptive feasibility analyses. All statistical analyses were performed in R version 4.5.2 (R Foundation for Statistical Computing, Vienna, Austria). A two-sided *p*-value of less than 0.05 was considered to indicate statistical significance.

## 3. Results

### 3.1. Baseline Characteristics and Participant Flow

**Figure 1:**
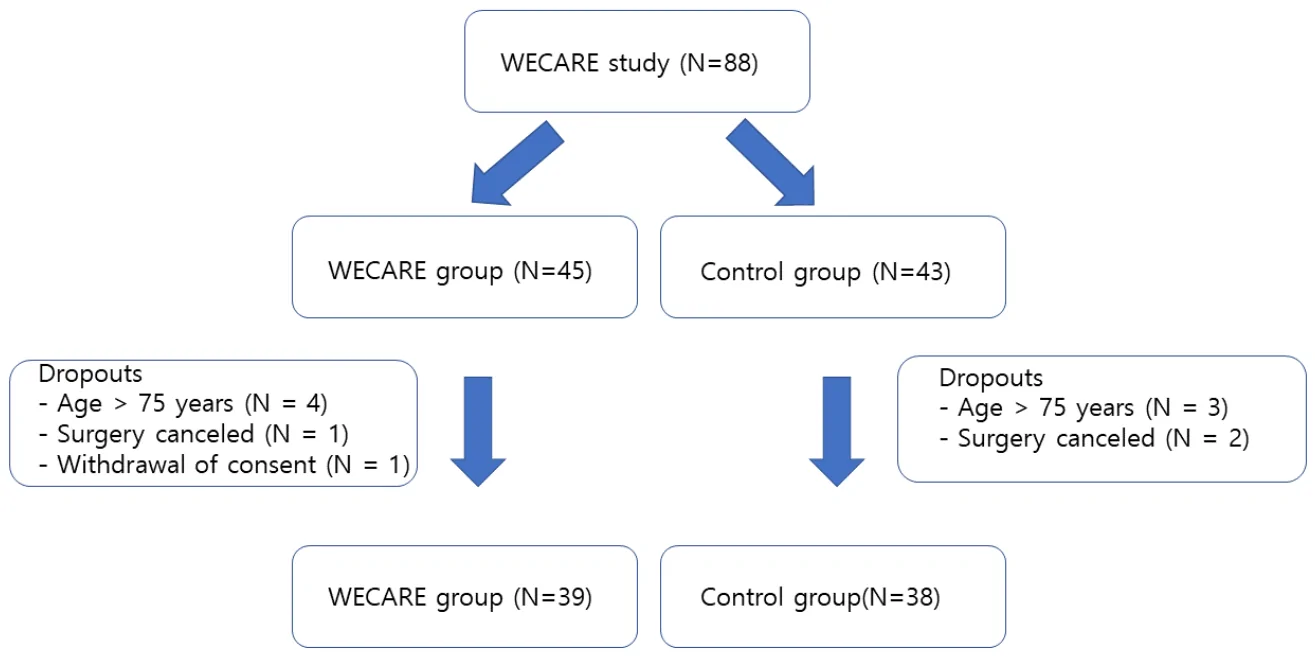
Flow diagram of patient enrollment, allocation, and follow-up.

A total of 88 patients were enrolled in this multicenter pilot randomized controlled trial (RCT), including 22 patients in the initial platform-optimization phase and 66 patients in the randomized phase (Figure 1). The mean age of the participants was 58.3 ± 10.7 years. The groups were generally well balanced in terms of baseline clinical and demographic variables, including body mass index (BMI) (23.9 vs. 23.2 kg/m^2^, *p* = 0.395) and comorbidities (*p* = 0.744). However, a statistically significant difference was observed in sex distribution, with the WECARE group having a greater proportion of female participants (46.7%) than the control group (25.6%, *p* = 0.04). Most patients underwent distal gastrectomy (WECARE: 66.7% vs. control: 67.4%), predominantly via laparoscopic or robotic approaches (Table 1).

### 3.2. Longitudinal Changes in Quality of Life (KOQUSS-40)

**Figure 2:**
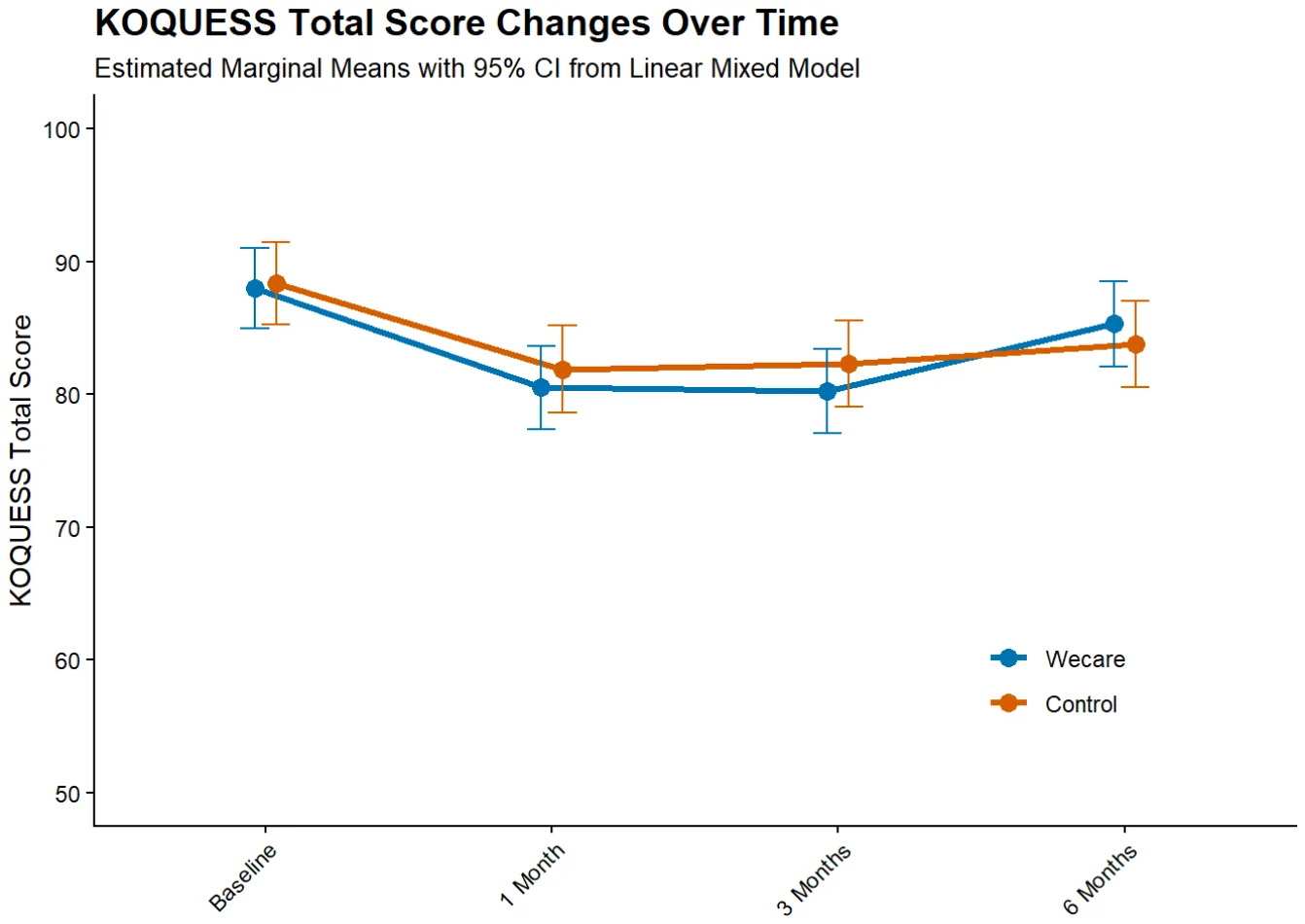
Longitudinal changes in KOQUSS-40 total scores over time. Estimated marginal means with 95% confidence intervals derived from a linear mixed-effects model are shown for the WECARE and control groups at baseline, 1, 3, and 6 months after surgery. The labeled trend lines illustrate the overall postoperative recovery pattern in both groups, with a numerically more favorable recovery trajectory in the WECARE group at 6 months. Higher scores indicate a better quality of life.

The primary outcome, the KOQUSS-40 total score, showed a characteristic recovery pattern following gastrectomy. At baseline, both groups had similar total scores (WECARE: 88 ± 1.5; control: 88.3 ± 1.6). After surgery, a decrease in QOL was observed in both groups at the 1-month and 3-month follow-ups, consistent with the expected early postoperative recovery course. At the 6-month follow-up, the WECARE group showed a numerically higher mean score (85.3 ± 1.6) than the control group (83.8 ± 1.6). Although the between-group difference at 6 months did not reach statistical significance (difference: 0.462; *p* = 0.603; Cohen’s d = −0.065), the longitudinal trajectory in the WECARE group appeared more favorable from the acute postoperative period onward (Table 2, Figure 2).

### 3.3. Subscale Analysis and Symptom-Specific Outcomes

**Figure 3:**
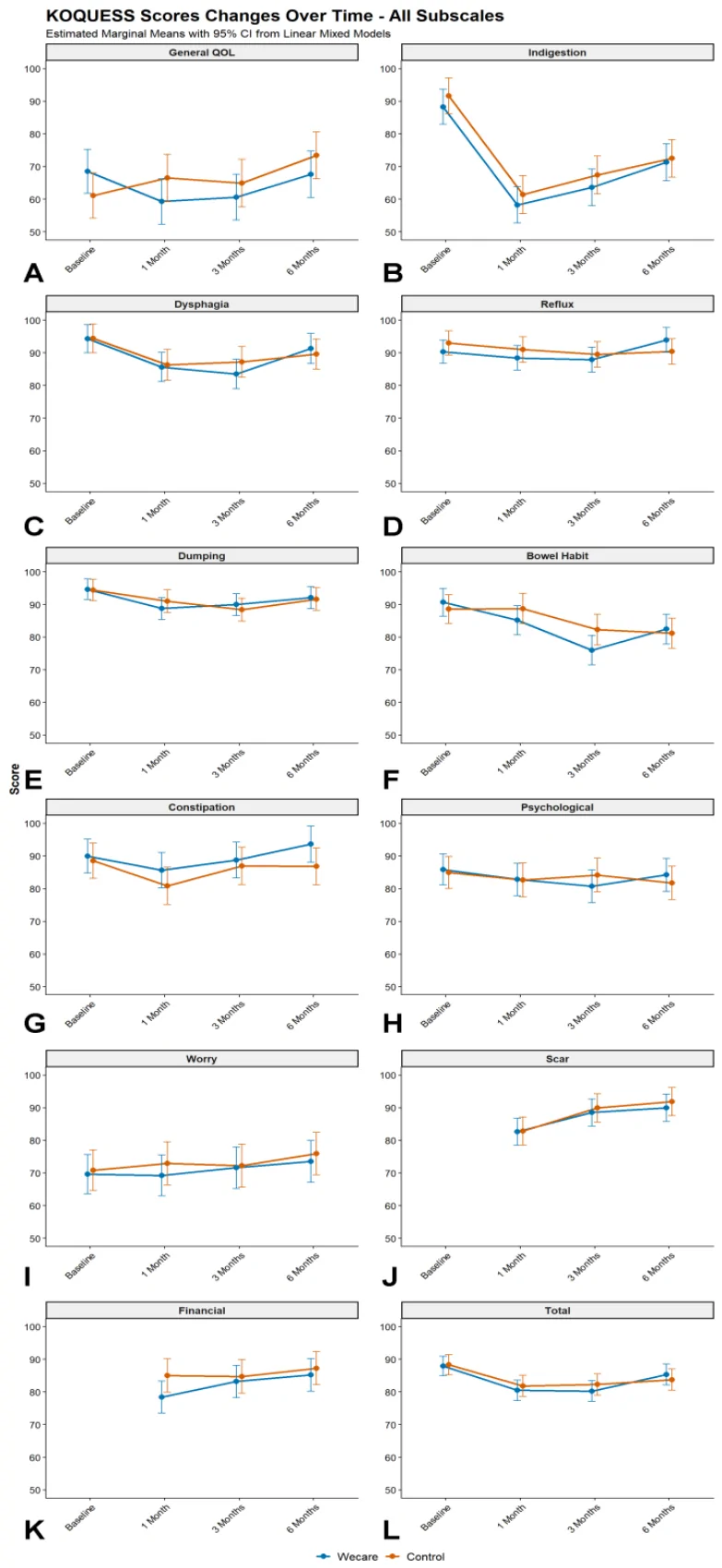
Longitudinal trends in KOQUSS-40 subscale scores; comparative analysis of recovery trajectories across seven functional domains between the WECARE and control groups over a 6-month postoperative period. (**A**) General QOL: assesses global subjective well-being. Differences observed at the 6-month mark should be interpreted cautiously because of baseline demographic imbalance, including sex distribution. (**B**) Indigestion, (**C**) Dysphagia, (**D**) Reflux: measures distress related to acid or bile reflux; the WECARE group showed a more favorable recovery trend than the control group, (**E**) Dumping syndrome: monitors the occurrence of early and late postprandial symptoms. (**F**) Bowel habit change, (**G**) Constipation (**H**) Psychological factors: captures emotional distress and anxiety associated with cancer survivorship. (**I**) Worry about cancer (**J**) Scar problem (**K**) Financial problem (**L**) Total score. All data are presented as estimated marginal means derived from a linear mixed-model analysis. Across most domains, a characteristic V-shaped recovery pattern was observed, characterized by an acute postoperative decline followed by gradual improvement.

Detailed subscale analysis revealed varying impacts of the digital intervention on specific domains of postgastrectomy syndromes. Most domains recovered comparably between the two groups. However, the “General QOL” subscale exhibited a statistically significant difference (*p* = 0.0403), with the control group reporting higher scores; this finding should be interpreted cautiously because of the baseline imbalance in sex distribution between the groups. By contrast, specific gastric symptoms such as reflux showed a positive trend toward improvement in the WECARE group (*p* = 0.0856) at 6 months. These findings suggest that although the overall score was not significantly altered, the platform may have supported symptom-specific recovery and self-management in selected domains (Figure 3).

### 3.4. Platform Adherence and Patient Satisfaction

The feasibility of the WECARE platform was further validated by high levels of patient engagement and satisfaction. Throughout the 6-month study period, the WECARE group maintained a high compliance rate for questionnaire completion (86.7%). After the final evaluation at 6 months, more than 82% of the users reported being “satisfied” or “very satisfied” with the platform (ratings of 5 or 6 on a 6-point scale). Specifically, 54% of the patients rated the app’s overall utility with the highest satisfaction score. In addition, 77% of the patients reported that the platform improved their self-management capabilities, and most participants (82%) indicated that they would recommend the platform to other gastric cancer survivors.

## 4. Discussion

This pilot randomized controlled trial showed that the WECARE platform is a feasible and acceptable digital intervention for gastric cancer survivors. Although the primary endpoint did not significantly differ between the two groups, the WECARE group exhibited a numerically more favorable recovery trajectory in overall quality-of-life (QOL) scores during the six-month follow-up period. Because this was a pilot multicenter trial, these findings should be interpreted as preliminary efficacy signals rather than definitive evidence of treatment effect. The high adherence rate (over 86%) and strong patient satisfaction nevertheless suggest that the platform can be integrated into postoperative recovery pathways and justify larger-scale validation.

A plausible explanation for the patient-perceived benefit of WECARE is not that the platform directly treated symptoms, but that it supported earlier recognition, structured reporting, and symptom-specific behavioral adjustment after gastrectomy. The KOQUSS-40-based interface may have helped patients better identify and communicate postoperative symptoms during follow-up, while algorithm-based feedback and educational content may have supported dietary adaptation, reflux and dumping precautions, physical activity, and self-management. In this regard, the bidirectional platform likely functioned as a guided survivorship support tool that reduced uncertainty and promoted more confident recovery behavior. Indeed, digital symptom monitoring for cancer patients has been reported to improve overall quality of life by reducing feelings of isolation and facilitating timely recognition of the need for clinical intervention [2,3,13,14,15,16]. Recent studies in gastrectomy populations have also suggested that mobile app-based coaching and digital therapeutics may improve symptom management, dietary adaptation, and patient engagement after surgery [17,18,19].

Furthermore, this pilot study provided a basis for refining next-generation platforms using real-world clinical data. The sex-based differences in baseline QOL-related characteristics and platform adherence patterns may provide useful signals for future interface tailoring. However, the present sample size was not sufficient for robust sex-stratified outcome inference, and sex-related observations should therefore be considered exploratory. Future adequately powered studies should evaluate whether symptom burden, engagement, and responsiveness to digital survivorship tools differ according to sex and other patient-level factors. These observations are also consistent with broader evidence showing that PRO-guided and ePRO-based interventions can improve quality of life, symptom control, and care delivery in oncology and survivorship settings [20,21,22,23,24,25].

Finally, beyond being a simple clinical tool, the WECARE platform demonstrates potential scalability and opportunities for broader implementation. Combining continuous glucose monitoring technology with dietary record data may help build models to predict and prevent dumping syndrome and hypoglycemia, which remain important challenges for gastric cancer survivors [26,27]. At the same time, the relatively small between-group difference observed in this study may partly reflect the high standard of postoperative care delivered at Korean tertiary referral centers. Therefore, future studies in broader healthcare settings, including international or less standardized survivorship-care environments, may provide a clearer perspective on the added clinical value and generalizability of this platform [9,28,29,30].

Despite the valuable insights gained from this pilot study, several limitations should be considered. First, the relatively small sample size may have limited the statistical power to detect differences in the primary endpoint. Second, the six-month follow-up period was designed to capture early postoperative recovery but may be insufficient to fully assess long-term adaptation and late complications after gastrectomy. Third, a baseline imbalance in sex distribution between the study groups may have influenced patient-reported outcomes, and the current sample size did not allow robust sex-stratified outcome analysis. Fourth, because postoperative recovery generally improves over time after gastrectomy, the numerically favorable 6-month pattern in the WECARE group cannot be attributed solely to the intervention and should instead be interpreted as a potentially more favorable recovery trajectory relative to standard care. Finally, the control group received high-quality standardized postoperative care at tertiary referral centers, which may have reduced the measurable incremental benefit of the digital intervention. Nevertheless, this study remains one of the first nationwide multicenter evaluations of a disease-specific digital platform for gastric cancer survivors and provides a practical basis for larger, longer, and more internationally generalizable studies.

## 5. Conclusions

WECARE was a feasible and well-accepted digital platform for postoperative survivorship care in patients with gastric cancer. Although the primary endpoint did not significantly differ between groups, patients using WECARE showed a numerically favorable recovery trajectory and high engagement with self-management. These findings support further evaluation of disease-specific digital symptom monitoring in larger multicenter trials with longer follow-up and broader healthcare settings.

## Figures and Tables

**Table 1:** Baseline Characteristics of the Study Population.

Characteristics	Overall (*n* = 88)	WECARE (*n* = 45)	Control (*n* = 43)	*p*-Value
Age (years)	58.3 ± 10.7	56.6 ± 11.1	60.1 ± 10.1	0.245
Sex				0.04
Male	56 (63.6%)	24 (53.3%)	32 (74.4%)	
Female	32 (36.4%)	21 (46.7%)	11 (25.6%)	
BMI (kg/m^2^)	23.6 ± 3.7	23.9 ± 3.8	23.2 ± 3.5	0.395
Comorbidity				0.744
none	58 (69.0%)	29 (67.4%)	29 (70.7%)	
≥1	26 (31.0%)	14 (32.6%)	12 (29.3%)	
Surgical approach				0.615
Open	1 (1.3%)	0 (0.0%)	1 (2.6%)	
Laparoscopy-assisted	3 (3.8%)	1 (2.4%)	2 (5.3%)	
Totally laparoscopic	68 (86.1%)	37 (90.2%)	31 (81.6%)	
Robot-assisted	7 (8.9%)	3 (7.3%)	4 (10.5%)	
Resection extent				0.285
Distal gastrectomy	65 (83.3%)	36 (87.8%)	29 (78.4%)	
Total gastrectomy	10 (12.8%)	3 (7.3%)	7 (18.9%)	
Proximal gastrectomy	2 (2.6%)	1 (2.4%)	1 (2.7%)	
Others	1 (1.3%)	1 (2.4%)	0 (0.0%)	
Reconstruction				0.841
Billroth I	5 (6.6%)	2 (5.1%)	3 (8.1%)	
Billroth II	39 (51.3%)	21 (53.8%)	18 (48.6%)	
Roux-en-Y (DG)	12 (15.8%)	7 (17.9%)	5 (13.5%)	
Roux-en-Y (TG)	13 (17.1%)	5 (12.8%)	8 (21.6%)	
Others	7 (9.2%)	4 (10.3%)	3 (8.1%)	
Curative resection				>0.999
R0	76 (98.7%)	38 (97.4%)	38 (100.0%)	
R2	1 (1.3%)	1 (2.6%)	0 (0.0%)	
Operation time (min)	179.0 ± 68.6	183.3 ± 70.4	174.3 ± 67.1	0.515
Blood loss (mL)	80.4 ± 113.1	90.6 ± 124.2	69.6 ± 100.8	0.359
Complication	6 (8.0%)	2 (5.3%)	4 (10.8%)	0.43
Clavien–Dindo grade				0.372
Grade 0	82 (93.2%)	43 (95.6%)	39 (90.7%)	
Grade I	3 (3.4%)	2 (4.4%)	1 (2.3%)	
Grade II	2 (2.3%)	0 (0.0%)	2 (4.7%)	
Grade III	1 (1.1%)	0 (0.0%)	1 (2.3%)	
Tumor location				
Upper	9 (11.5%)	5 (12.5%)	4 (10.5%)	>0.999
Middle	25 (32.1%)	9 (22.5%)	16 (42.1%)	0.064
Lower	45 (57.7%)	27 (67.5%)	18 (47.4%)	0.072
AJCC stage				0.157
IA	39 (52.7%)	20 (51.3%)	19 (54.3%)	
IB	12 (16.2%)	3 (7.7%)	9 (25.7%)	
IIA	6 (8.1%)	4 (10.3%)	2 (5.7%)	
IIB	10 (13.5%)	5 (12.8%)	5 (14.3%)	
IIIA	2 (2.7%)	2 (5.1%)	0 (0.0%)	
IIIB	2 (2.7%)	2 (5.1%)	0 (0.0%)	
IIIC	1 (1.4%)	1 (2.6%)	0 (0.0%)	
Unknown	2 (2.7%)	2 (5.1%)	0 (0.0%)	

Values are presented as mean ± SD or number (%); *p*-values were calculated using the Wilcoxon rank-sum test, chi-square test, or Fisher’s exact test as appropriate.

**Table 2:** Longitudinal changes in KOQUSS-40 total scores based on a linear mixed-effects model.

Time Point	WECARE Group	Control Group	Difference	*p*-Value
Baseline	88 ± 1.5	88.3 ± 1.6		
1 Month	80.5 ± 1.6	81.9 ± 1.7		
3 Months	80.3 ± 1.6	82.3 ± 1.7		
6 Months	85.3 ± 1.6	83.8 ± 1.6	0.462	0.603

Model-based estimates: Slope (per month): WECARE −0.931 ± 0.618; Control −1.393 ± 0.635; Between-group difference at 6 months: 0.462 (*p* = 0.603); Effect size (Cohen’s d): −0.065; Values are presented as estimated marginal means ± standard error (standard deviation). Slope represents the average monthly change in KOQUSS-40 total score. Between-group difference is defined as WECARE minus Control at 6 months. Cohen’s d interpretation: <0.2 negligible, 0.2–0.5 small, 0.5–0.8 moderate, ≥0.8 large. Higher scores indicate a better quality of life.

## Data Availability

The data that support the findings of this study are available from the corresponding author upon reasonable request.
